# Conduct and reporting of individual participant data network meta-analyses need improvement

**DOI:** 10.1186/s12916-020-01630-w

**Published:** 2020-06-02

**Authors:** Anna Chaimani

**Affiliations:** 1 Université de Paris, Research Center of Epidemiology and Statistics (CRESS-U1153), INSERM, INRA, F-75004 Paris, France; 2 Cochrane France, Paris, France

**Keywords:** Multiple treatments, Evidence synthesis, Comparative effectiveness research, Patient-level information, Critical appraisal

## Background

Network meta-analysis (NMA) of healthcare interventions is being increasingly used in medical research aiming to address a key question for clinical decision making: *which interventions work best for a given disease?* The popularity of NMA builds on three main characteristics that make it a unique tool in the field of evidence synthesis: (a) it allows inference for comparisons that have never been evaluated in individual studies, (b) it usually gives relative effect estimates with the highest precision, and (c) it allows estimating the ranking of interventions with respect to some outcomes of interest [1, 2]. Despite these benefits, a major constraint of most NMAs to date is that they are primarily considering aggregate data, hence data at study level or, at best, arm-level data.

The use of individual participant data (IPD) in meta-analyses is known to have several advantages, such as allowing to handle properly missing data, investigation of associations between outcomes and participants’ characteristics, and exploration of case-mix heterogeneity [3]. According to the article by Gao et al., though, only 21 NMAs of randomized controlled trials (RCTs) with available IPD were published until June 2019 [4]. On the contrary, a collection of NMAs with aggregate RCT data included, already in 2015, 450 publications [5]; this number has probably been tripled now. The main explanation of this imbalance is probably the difficulties encountered in obtaining IPD from trial investigators. Generally, IPD sharing is a rather time- and resource-consuming procedure and, until recently, was restricted within small research communities. On top of data availability issues, conducting NMA with IPD requires knowledge of advanced statistical modeling approaches and specific expertise in the review team. This increased complexity is sometimes an obstacle for NMA investigators leading them to refrain from an IPD analysis plan.

## Recent advances

Interestingly, the study by Gao et al. does not show a consistent increase in the publications of IPD NMAs over years as it would be expected given the overall steep increase of NMAs in the literature [5]. However, new initiatives, such as YODA (https://yoda.yale.edu/) and Clinical Study Data Request (https://www.clinicalstudydatarequest.com/), that aim to promote large-scale IPD sharing will possibly facilitate the access of meta-analysts to such data. At the same time, researchers have started being more familiar with methods for incorporating IPD in NMA due to the greater focus placed lately in the field including new developments [6, 7] and several training events (e.g., Cochrane webinars). Considering these important advances, a larger number of IPD NMAs is anticipated to be published in the next few years. It is of great importance, though, to reassure that they will follow high-quality standards, since they are usually considered superior to NMAs of aggregate data and their conclusions may have stronger impact.

## Empirical results

Gao et al. found important deficiencies in the 21 IPD NMAs they identified. Specifically, they evaluated the methodological and reporting quality of these NMAs using three tools: the PRISMA-IPD [8], the PRISMA-NMA [9], and the AMSTAR-2 [10] checklists (after removing overlapping items) and concluded that the overall quality of the publications was suboptimal. One of the most important limitations was the lack of an assessment of the NMA assumptions. None of the articles reported assessment of the fundamental conceptual synthesis assumption and less than half assessed the respective statistical assumption. Further, most of the NMAs ignored missing data or used naïve imputation methods, none described how IPD and aggregate data were combined when both were used, and only 10% reported registration of a protocol and evaluation of across-study biases. Based on AMSTAR-2, the majority of these NMAs were rated as being of critically low quality. It is encouraging that involvement of a statistician or epidemiologist in the review team was associated with overall better methodological and reporting quality.

These findings raise concerns with respect to the validity of results from published IPD NMAs. Of course, some of these articles were published before the development of the appraisal tools that were used and full compliance could not be expected. Deficiencies, though, did not seem to be mitigated after the development of the checklists. For example, PRISMA-NMA clearly states that evaluation of the synthesis assumptions is crucial, but even subsequent IPD NMAs did not follow this recommendation. It is also possible that some of the identified deficiencies are the result of poor reporting and do not necessarily show poor methodological quality.

## Conclusions

In conclusion, the study by Gao et al. poses an important question: whether existing guidelines for conducting and reporting NMAs in the presence of IPD are sufficient. Although using a combination of PRISMA-IPD and PRISMA-NMA would cover most of the required information that needs to be reported, it seems reasonable to move towards the development of specific guidance for IPD NMAs. The development of a comprehensive list of items that any IPD NMA should report, such as an extension of the PRISMA-NMA, would be helpful not only to NMA authors but also to journal editors and reviewers who will be able to easily judge the reporting and methodological completeness of these articles.

## Data Availability

Not applicable

## Abbreviations

AMSTAR: A MeaSurement Tool to Assess systematic Reviews
IPD: Individual participant data
NMA: Network meta-analysis
PRISMA: Preferred Reporting Items for Systematic Reviews and Meta-Analyses
RCT: Randomized controlled trial
YODA: Yale University Open Data Access
